# Supplementary material for: Low-energy electronic structure in the unconventional charge-ordered state of ScV6Sn6
Source: Nat Commun. 2024 Jun 12;15:5008. doi: 10.1038/s41467-024-48883-0 (PMC11169263; doi:10.1038/s41467-024-48883-0)
Supplement: Supplementary file 1 — Supplementary Information [file 41467_2024_48883_MOESM1_ESM.pdf]

Supplementary Information for "Low-Energy Electronic Structure in the Unconventional Charge-Ordered State of ScV_6Sn_6"

Asish K. Kundu[1†], Xiong Huang[2†], Eric Seewald[2], Ethan Ritz[3], Santanu Pakhira[4,5], Shuai Zhang[2], Dihao Sun[2], Simon Turkel[2], Sara Shabani[2], Turgut Yilmaz[6], Elio Vescovo[6], Cory R. Dean[2], David C. Johnston[4,7], Tonica Valla[8], Turan Birol[3], Dmitri N. Basov[2], Rafael M. Fernandes[9] and Abhay N. Pasupathy[1,2*]

[1]Condensed Matter Physics and Materials Science Division, Brookhaven National Laboratory, Upton, NY 11973, USA.
[2]Department of Physics, Columbia University, New York, NY 10027, USA.
[3]Department of Chemical Engineering and Materials Science, University of Minnesota, Minneapolis, MN 55455, USA.
[4]Ames National Laboratory, Iowa State University, Ames, Iowa 50011, USA.
[5]Present Address: Institute for Quantum Materials and Technologies, Karlsruhe Institute of Technology, Karlsruhe, D-76021, Germany.
[6]National Synchrotron Light Source II, Brookhaven National Laboratory, Upton, NY 11973, USA.
[7]Department of Physics and Astronomy, Iowa State University, Ames, Iowa 50011, USA.
[8]Donostia International Physics Center (DIPC), 20018 Donostia-San Sebastián, Spain.
[9]School of Physics and Astronomy, University of Minnesota, Minneapolis, MN 55455, USA.

*Corresponding author(s). E-mail(s): apn2108@columbia.edu;
†These authors contributed equally to this work.

1 Supplementary Information

1.1 CDW Lattice Distortion and Its Coupling to the Electronic Spectrum

Here we review the electronic properties of a generic single-band system with electronic dispersion $\xi_{\mathbf{k}}$ in the presence of CDW order with wave-vector $\mathbf{K}_{\mathrm{CDW}}$. Using standard many-body methods [1], the retarded Green's function inside the CDW phase can be written as: $G^{-1} = G_0^{-1} - \Sigma$, where $G_0^{-1} = \omega - \xi_{\mathbf{k}}$ is the Green's function in the disordered state and the self-energy is given by $\Sigma = \frac{\lambda^2 \Delta^2}{\omega - \xi_{\mathbf{k}+\mathbf{K}_{\mathrm{CDW}}}}$. Here, λ and Δ are the coupling constant and order parameter associated with the lattice distortion, respectively. Thus, G^{-1} can be written as:

$$\begin{aligned} G^{-1}(\omega, \mathbf{k}) &= \omega - \xi_{\mathbf{k}} - \frac{\lambda^2 \Delta^2}{\omega - \xi_{\mathbf{k}+\mathbf{K}_{\mathrm{CDW}}}} \\ &= \frac{(\omega - E_{+,\mathbf{k}})(\omega - E_{-,\mathbf{k}})}{\omega - \xi_{\mathbf{k}+\mathbf{K}_{\mathrm{CDW}}}} \end{aligned} \tag{1}$$

where, as usual for retarded functions, ω should be understood as $\omega + i0^+$, and we defined:

$$E_{\pm,\mathbf{k}} \equiv \left(\frac{\xi_{\mathbf{k}} + \xi_{\mathbf{k}+\mathbf{K}_{\mathrm{CDW}}}}{2} \right) \pm \sqrt{\left(\frac{\xi_{\mathbf{k}} - \xi_{\mathbf{k}+\mathbf{K}_{\mathrm{CDW}}}}{2} \right)^2 + \lambda^2 \Delta^2} \tag{2}$$

The Green's function has poles at $E_{\pm,\mathbf{k}}$, which correspond to the standard expressions for the reconstructed electronic dispersion inside the CDW phase. The dispersion can be obtained by superimposing the unfolded band $\xi_{\mathbf{k}}$ and

the folded band $\xi_{\mathbf{k}+\mathbf{K}_{\rm CDW}}$, with a gap proportional to $\lambda\Delta$ opening at the intersections between them.

The ARPES and QPI spectra are determined not only by the reconstructed electronic dispersion, but also by its spectral weight, which itself depends on the coupling constant λ as well. To see this, we rewrite Eq. (1) as:

$$G\left(\omega,\mathbf{k}\right)=\frac{W_{+,\mathbf{k}}}{\omega-E_{+,\mathbf{k}}}+\frac{W_{-,\mathbf{k}}}{\omega-E_{-,\mathbf{k}}} \tag{3}$$

where $W_{\pm}$, the spectral weights of the poles, are given by:

$$W_{\pm,\mathbf{k}}=\frac{1}{2}\left(1\pm\frac{\mathrm{sgn}\left(\xi_{\mathbf{k}}-\xi_{\mathbf{k}+\mathbf{K}_{\rm CDW}}\right)}{\sqrt{1+\left(\frac{2\lambda\Delta}{\xi_{\mathbf{k}}-\xi_{\mathbf{k}+\mathbf{K}_{\rm CDW}}}\right)^2}}\right) \tag{4}$$

To proceed, we define $\delta_{\mathbf{k}}\equiv\mid\xi_{\mathbf{k}}-\xi_{\mathbf{k}+\mathbf{K}_{\rm CDW}}\mid/2$ and consider any momenta $\mathbf{k}$ for which $\delta_{\mathbf{k}}\gg\lambda\Delta$. For small coupling λ, this condition is met for a wide range of momenta for which the folded and unfolded bands do not cross. Expanding in $\lambda\Delta/\delta_{\mathbf{k}}$ then gives:

$$\begin{aligned}E_{\mathrm{sgn}(\delta),\mathbf{k}}&\approx\xi_{\mathbf{k}}\\E_{-\mathrm{sgn}(\delta),\mathbf{k}}&\approx\xi_{\mathbf{k}+\mathbf{K}_{\rm CDW}}\\W_{\mathrm{sgn}(\delta),\mathbf{k}}&\approx1-\frac{\lambda^2\Delta^2}{4\delta_{\mathbf{k}}^2}\\W_{-\mathrm{sgn}(\delta),\mathbf{k}}&\approx\frac{\lambda^2\Delta^2}{4\delta_{\mathbf{k}}^2}\end{aligned} \tag{5}$$

such that the Green's function inside the CDW phase becomes:

$$G\left(\omega,\mathbf{k}\right)\approx\left(1-\frac{\lambda^2\Delta^2}{4\delta_{\mathbf{k}}^2}\right)\frac{1}{\omega-\xi_{\mathbf{k}}}+\left(\frac{\lambda^2\Delta^2}{4\delta_{\mathbf{k}}^2}\right)\frac{1}{\omega-\xi_{\mathbf{k}+\mathbf{K}_{\rm CDW}}} \tag{6}$$

Clearly, for these momenta values, the spectral weight of the folded bands is negligible. Since ARPES directly measures the electronic spectral weight $A(\omega, \mathbf{q}) = -\frac{1}{\pi}\mathrm{Im}\, G(\omega, \mathbf{q})$, the folded bands are not expected to be visible in this regime. As for the QPI signal $\delta n(\omega, \mathbf{q})$, it depends not only on the electronic spectral weight, but also on the impurity potential encoded in the T-matrix:

$$\delta n(\omega, \mathbf{q}) = -\frac{1}{\pi}\mathrm{Im} \int G(\omega, \mathbf{k}) T(\omega;\ \mathbf{k}, \mathbf{k}+\mathbf{q}) G(\omega, \mathbf{k}+\mathbf{q})\, d\mathbf{k} \tag{7}$$

Using Eq. (6), it is clear that for a featureless T-matrix, $T(\omega;\ \mathbf{k}, \mathbf{k}+\mathbf{q}) = T_0(\omega)$, corresponding e.g. to a point-like impurity potential, the QPI inside the CDW ordered state is well approximated by that of the disordered phase, $\delta n(\omega, \mathbf{q}) \approx \delta n_0(\omega, \mathbf{q}) + \mathcal{O}(\lambda^2\Delta^2)$, where we defined

$$\delta n_0(\omega, \mathbf{q}) = -\frac{1}{\pi}\, T_0(\omega)\, \mathrm{Im} \int G_0(\omega, \mathbf{k}) G_0(\omega, \mathbf{k}+\mathbf{q})\, d\mathbf{k} \tag{8}$$

The folded bands could however become visible in the QPI signal if the T-matrix was not featureless, but had a component that is strongly peaked at the CDW wave-vector $\mathbf{K}_{\mathrm{CDW}}$, thus connecting both the folded and unfolded bands.

To further illustrate the effect of the coupling constant λ on the ARPES and QPI spectra, we consider a nearest neighbor tight-binding model on a triangular lattice. The energy dispersion is given by

$$\xi_{\mathbf{k}} = -2t + 4t\cos(k_x a/2)\left[\cos(k_x a/2) + \cos(\sqrt{3}k_y a/2)\right] \tag{9}$$

where t is the hopping parameter. We then impose a CDW with a wave-vector of $\mathbf{K}_{\mathrm{CDW}} = (1/3, 1/3)$, similar to the case of $ScSn_6V_6$. We vary the coupling constant λ according to $\frac{\lambda\Delta}{t} = 0, 0.05, 0.1, 0.2, 0.5$.

We show in SI Fig. 11**a-e** the spectral function (which ARPES directly probes) along the k_x direction plotted in units of $1/t$ for the various choices of the coupling constant. We used Eq. (1) to make these plots. Clearly, as λ increases, both the intensity of the folded bands and the gaps increase. In SI Fig. 11**f-j**, we show the corresponding Fermi surface, which consists of a six-fold symmetric Fermi pocket centered at the Γ point in the Brillouin zone (for a specific choice of the chemical potential, $\mu = 1.4t$, and lifetime is $4 \times 10^{-5}t$). Once again, the folded bands become more evident as the coupling constant increases.

To model the QPI, we calculate the JDOS, i.e. the autocorrelation of the spectral function in (1) at the Fermi level. The result is plotted in SI Fig. 11**k-o**. While there are several features in the JDOS, the relevant scattering vectors are those that are close to the CDW wave-vector, corresponding to scattering between the unfolded and folded bands. A zoom-in of the JDOS around one of the CDW wave-vectors is shown in SI Fig. 11**p-t**. It is clear that the intensity of the scattering between the unfolded and folded bands increases proportionally with the spectral weight of the folded bands.

1.2 Quasiparticle Interference versus the Joint Density of States

In general, Quasiparticle interference (QPI) and the Joint Density of States (JDOS) are distinct mathematical quantities. Within perturbation theory, under the assumption of a featureless T-matrix,

$$\mathrm{JDOS}(\mathbf{q}) \propto \int \mathrm{Im}(G_0(\omega, \mathbf{k}))\mathrm{Im}(G_0(\omega, \mathbf{k}+\mathbf{q}))d\mathbf{k}$$

while

$$\text{QPI}(\mathbf{q}) \propto \text{Im} \int G_0(\omega, \mathbf{k}) G_0(\omega, \mathbf{k} + \mathbf{q}) d\mathbf{k}$$

where $G_0(\omega, \mathbf{k})$ is the Green's function. In our specific case, we are concerned with scattering vectors $\mathbf{q}$ that are connect the unfolded and folded bands - ie, one of the Green's functions in the integral belongs to the original (normal state) band, while the other Green's function arises from band folding. In general, when the spectral intensity of the folded band is weak (as evidenced by ARPES), we expect that both the real and imaginary part of the Green's function from the folded band is also weak (see discussion in the preceeding section). Thus, even though the JDOS and QPI are generally different quantities, we expect the intensity of the scattering from the folded bands to be weak in both quantities in our case. The JDOS is however a much simpler quantity to calculate directly from spectral functions.

As an aside, in previous research on the CsV_3Sb_5 compound [2] which shares a very similar Fermi surface to ScV_6Sn_6, the JDOS nicely explains the main features of the experimentally observed QPI.

1.3 Momentum dependence of the T-Matrix

Our experimental data requires the presence of a T-matrix with strong momentum dependence. To consider how such a T matrix can arise in a solid, let us first consider a simple one-dimensional lattice model with a defect that scatters electrons between eigenstates. At the single particle level, this defect will create a scattering potential $V(r)$. To the lowest order, the T matrix is simply the Fourier transform of the real space potential $V(r)$. The commonly used potential in QPI calculations is a delta function impurity:

$$V(r) = \delta(r)$$

This potential gives a T matrix that is constant in reciprocal space, as is well known.

Now consider a different potential in real space:

$$V(r) = e^{-\xi|x|}cos(Gx)$$

Here ξ is a decay constant, and G is the reciprocal lattice vector. This potential is oscillatory and has the underlying periodicity of the lattice. Such a potential can arise very naturally in a solid - for example, a charge placed on one atom can cause a response on several atoms that are nearby, with a response that gradually decays at large distances. The T matrix corresponding to this real space potential is

$$T(k) = \frac{1}{\sqrt{2\pi}}(\frac{\xi}{\xi^2 + (k-G)^2} + \frac{\xi}{\xi^2 + (k+G)^2})$$

As is evident, this T matrix is peaked at the reciprocal lattice vector and will cause maximal scattering between states separated by this wave vector.

The generalization to the CDW case is straightforward. Simply replacing G by G_{CDW} gives a T matrix that is peaked at the CDW wave vector, as desired. Such a potential in real space can either come from a static enhancement of the CDW in the vicinity of a defect or from dynamical CDW fluctuations near the defect.

References

[1] Altland, A., Simons, B.D.: Condensed Matter Field Theory, 2nd edn. Cambridge University Press, ??? (2010)

[2] Zhao, H., Li, H., Ortiz, B.R., Teicher, S.M., Park, T., Ye, M., Wang, Z., Balents, L., Wilson, S.D., Zeljkovic, I.: Cascade of correlated electron states in

the kagome superconductor CsV_3Sb_5. Nature **599**(7884), 216–221 (2021)

[3] Hu, Y., Wu, X., Yang, Y., Gao, S., Plumb, N.C., Schnyder, A.P., Xie, W., Ma, J., Shi, M.: Tunable topological Dirac surface states and van Hove singularities in kagome metal GdV_6Sn_6. Science Advances **8**(38), 2024 (2022)

[4] Hu, Y., Ma, J., Li, Y., Gawryluk, D.J., Hu, T., Teyssier, J., Multian, V., Yin, Z., Jiang, Y., Xu, S., et al.: Phonon promoted charge density wave in topological kagome metal ScV_6Sn_6. arXiv preprint arXiv:2304.06431 (2023)

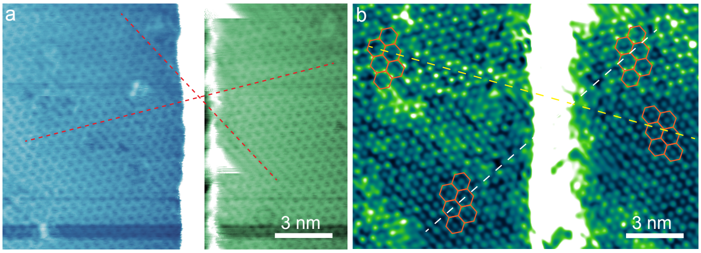

Supplementary Fig. 1: CDW modulation across a single-unitcell step ($\sim 0.9\,nm$). **a**,**b**, Topography at (25 mV, 100 pA) and the corresponding DOS image with a 5 mV bias modulation applied. The hexagons and dashed lines are used to demonstrate the $2\pi/3$ phase shift along two unidirectional CDW propagating directions. Near this single-unitcell step edge, both terraces are V-terminated surface. The result unambiguously demonstrates a 3D CDW order.

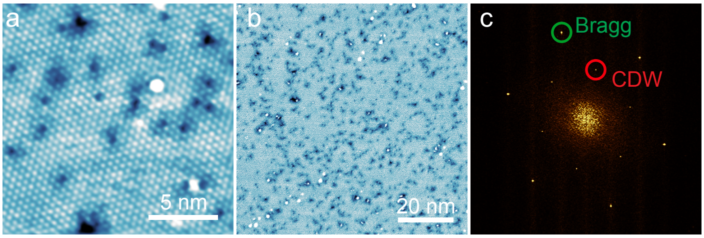

Supplementary Fig. 2: STM topography on the $ScSn_2$ surface termination.a, STM image of the upper terrace at a step ($\sim$ 0.18 nm) showing a triangular lattice (-50 mV, 120 pA). While the lower terrace shows a V-terminated surface, this triangular surface points to the $ScSn_2$ terminated surface. **b**, A large size topography (-100 mV, 190 pA). **c**, FFT of (b) showing CDW peaks (red circle) besides the Bragg peaks (green circle).

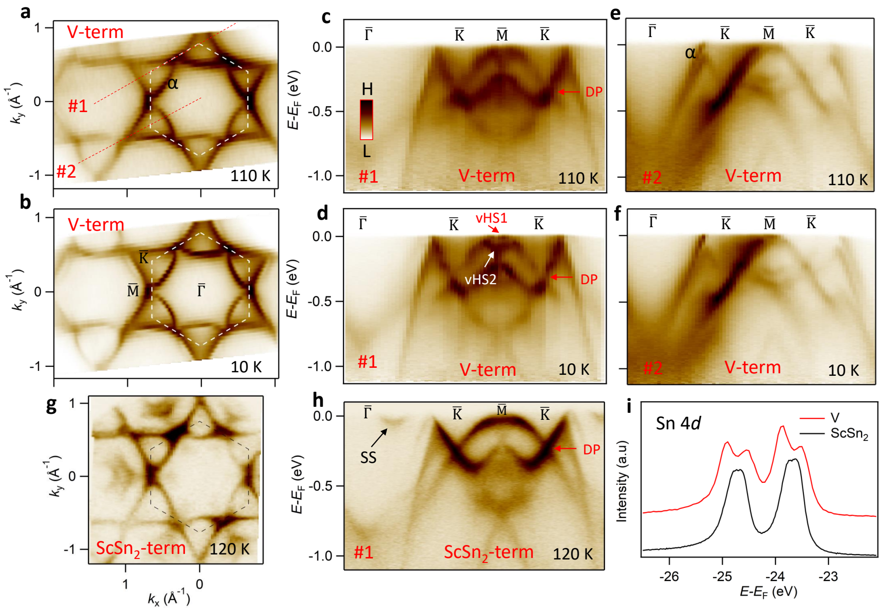

Supplementary Fig. 3: Electronic structure comparison between V- and $ScSn_2$-terminated surface. a and **b**, Fermi surface map above and below the CDW phase transition from V-terminated surface using $h\nu = 135$ eV. **c** and **d**, Band dispersion along a high symmetry direction as shown by line 1 in (a), above and below CDW transitions, respectively. **e** and **f**, Same as **c** and **d** but along line 2. **g**, Fermi surface map above CDW transition from $ScSn_2$-terminated surface using $h\nu = 140$ eV. (h) Band dispersion along line 1 as shown in **a** but for the $ScSn_2$-terminated surface. On the $ScSn_2$-terminated surface, additional Fermi surfaces are observed around the center of the second Brillouin zone due to the presence of surface states (indicated in **h**), compared to the V-terminated surface, similar to the previous report [3, 4]. These states are not seen in the 1st BZ because of the strong photoemission matrix element effects. **i** Sn 4d core level spectra from V- and $ScSn_2$-termination. Spectral feature differences between these two surfaces suggest different local environments of Sn atoms [4].

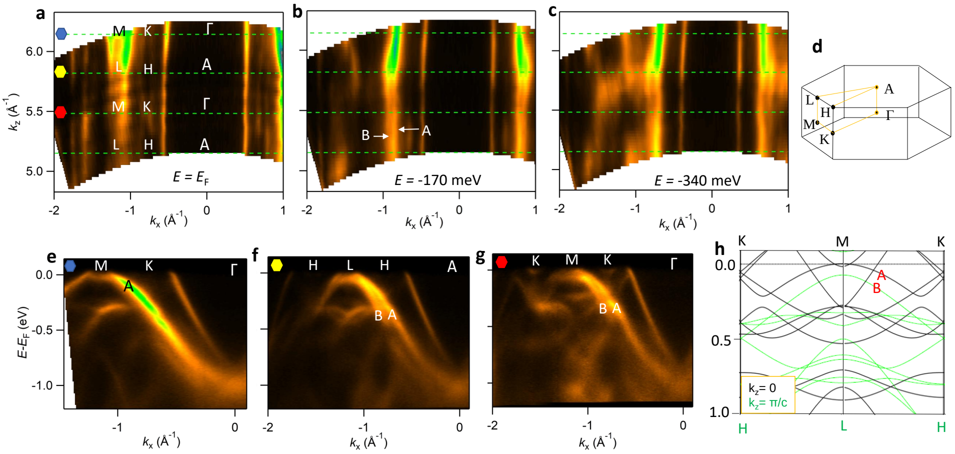

Supplementary Fig. 4: Photon-energy dependent electronic structure of ScV_6Sn_6. **a**, Fermi surface map in the k_x–k_z plane within a wide range of photon energies (87–140 eV) at T = 120 K. Brillouin zone high symmetry points were determined using $c = 9.17$ Å with an empirical inner potential of 14 eV. **b** and **c**, Same as **a** but with cuts at $E = -170$ meV and $E = -340$ meV, respectively. **d**, Schematics of the 3D BZ. **e**–**g**, Band dispersion for various k_z cuts as indicated in **a** by different color symbols. **h**, Theoretical bands along KMK and HLH paths are superimposed. Bands with similar dispersion are marked by A and B in **f**–**h**. Bands A and B coexist both at k_z= 0 (**g**) and k_z= π/c (**f**), suggests strong k_z broadening effects.

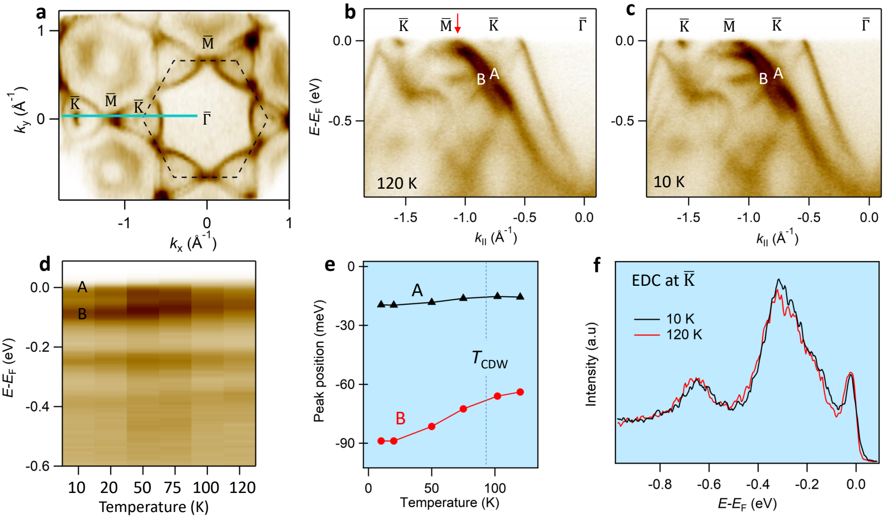

Supplementary Fig. 5: Temperature dependent electronic structure of ScV_6Sn_6. a, Fermi surface map from $ScSn_2$-terminated surface using $h\nu =$ 135 eV. **b** and **c**, Band dispersion, slightly off from the $\bar{\Gamma}$-$\bar{K}$-$\bar{M}$-$\bar{K}$ line as indicated by solid-line in **a** at 120 K and 10 K, respectively. Bands indicated by A and B in panel **c** and **d** appear from the $k_z = 0$ and $k_z = \pi/c$ planes, respectively (see Supplementary Fig. 4). The band A is forming the vHS1 at $\bar{M}$ point. **d**, The temperature dependence of electronic states along a cut as indicated by the arrow in **b**. (e), Change in the energy position of A and B bands with temperatures at momentum indicated by arrow in **b**. The position of the A band is nearly constant across the transition, while the B band is shifting to a higher binding energy below CDW. **f**, EDC at $\bar{K}$ across CDW transition does not show significant difference.

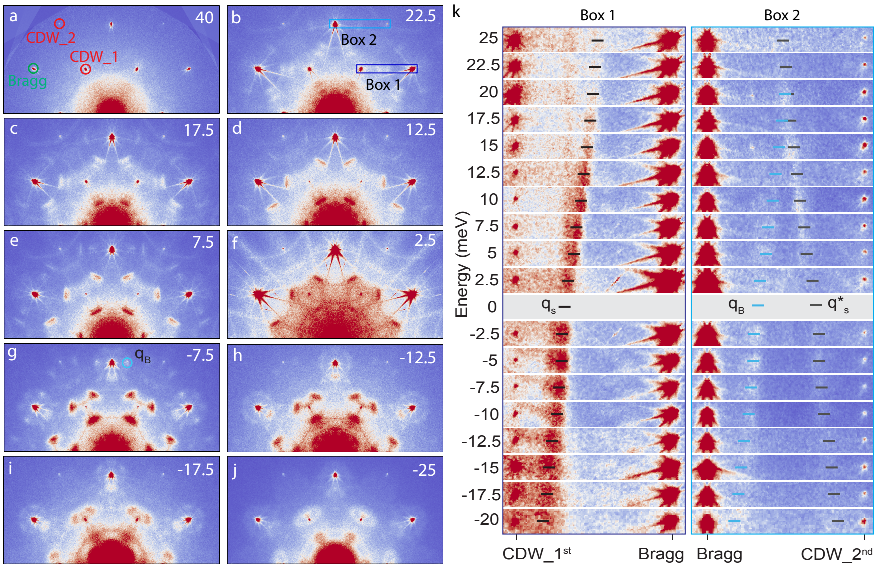

Supplementary Fig. 6: Additional QPI data on the V-layer. a-j, Symmetrized QPI images at different energies, respectively. In (g), the QPI wavevector (q_B) around the Bragg peak is highlighted by the light blue circle, which corresponds to scattering vectors between normal state hot-spots of the α band. **k**, Zoom in images (Box 1 and 2 in (b)) display the dispersion of q_s, q_B and $q^*{}_s$. $q^*{}_s$ is the replica of q_s in the second Brillouin zone. We note that straight line features in (f) are due to a line artifact in the raw FT data. STM setup (V_{sample}, I_{set}, V_{exc},) conditions for each panel: **a**, (40 mV, 140 pA, 2 mV); **b**, (22.5 mV, 120 pA, 2 mV); **c**, (17.5 mV, 120 pA, 2 mV); **d**, (12.5 mV, 110 pA, 1.5 mV); **e**, (7.5 mV, 110 pA, 1.5 mV); **f**, (2.5 mV, 80 pA, 1 mV); **g**, (−7.5 mV, 125 pA, 1 mV); **h**, (−12.5 mV, 130 pA, 1 mV); **i**, (−17.5 mV, 135 pA, 1.5 mV); **j**, (−25 mV, 145 pA, 2.5 mV).

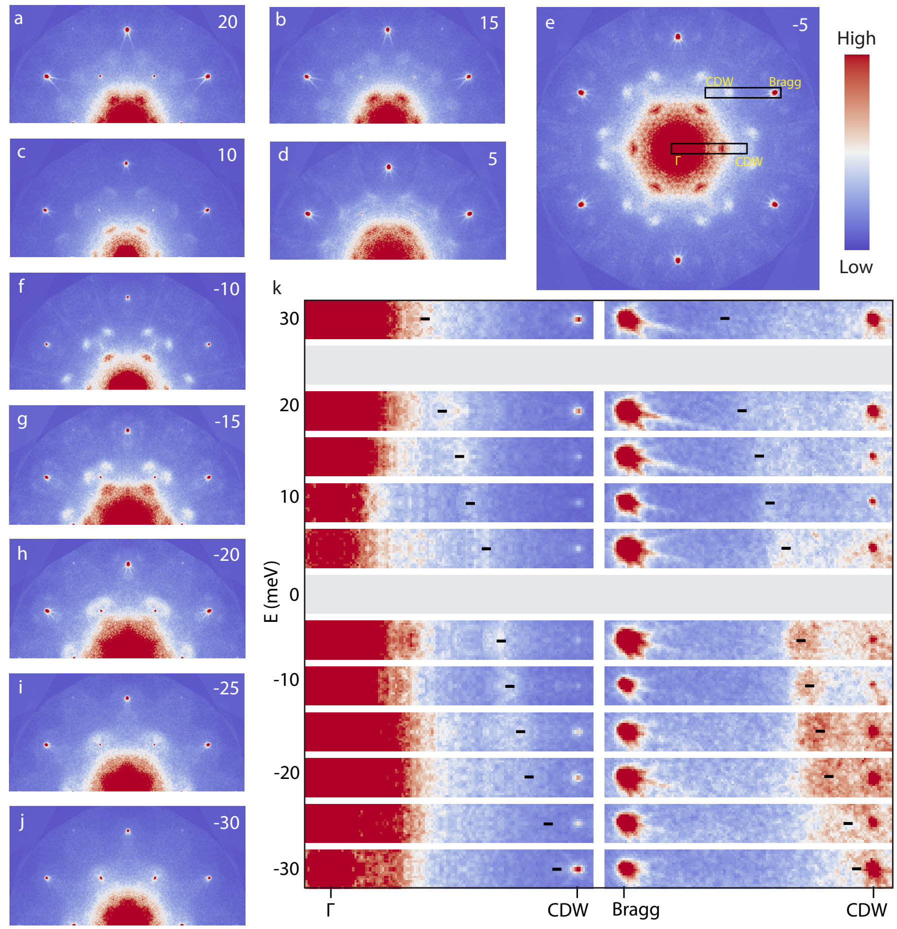

Supplementary Fig. 7: QPI maps on the $ScSn_2$-terminated surface. a-j, Experimental QPI maps at different energies after symmetrization on the $ScSn_2$ surface. Prominent QPI spots surrounding the CDW peaks disperse toward the CDW at lower energy and merge together at around -30 meV. **k**, The obtained dispersion for QPI vectors are consistent with those obtained on the vanadium surface. The real space images for all panels were acquired over a 100 nm field of view. STM setup (V_{sample}, I_{set}, V_{exc},) conditions for each panel: (30 mV, 150 pA, 6.5 mV); (20 mV, 150 pA, 4.5 mV); (15 mV, 140 pA, 3.5 mV); (10 mV, 120 pA, 2 mV); (5 mV, 80 pA, 2 mV); (−5 mV, 120 pA, 2 mV); (−10 mV, 150 pA, 2 mV); (−15 mV, 160 pA, 4 mV); (−20 mV, 170 pA, 5 mV); (−25 mV, 170 pA, 5 mV); (−30 mV, 170 pA, 5 mV).

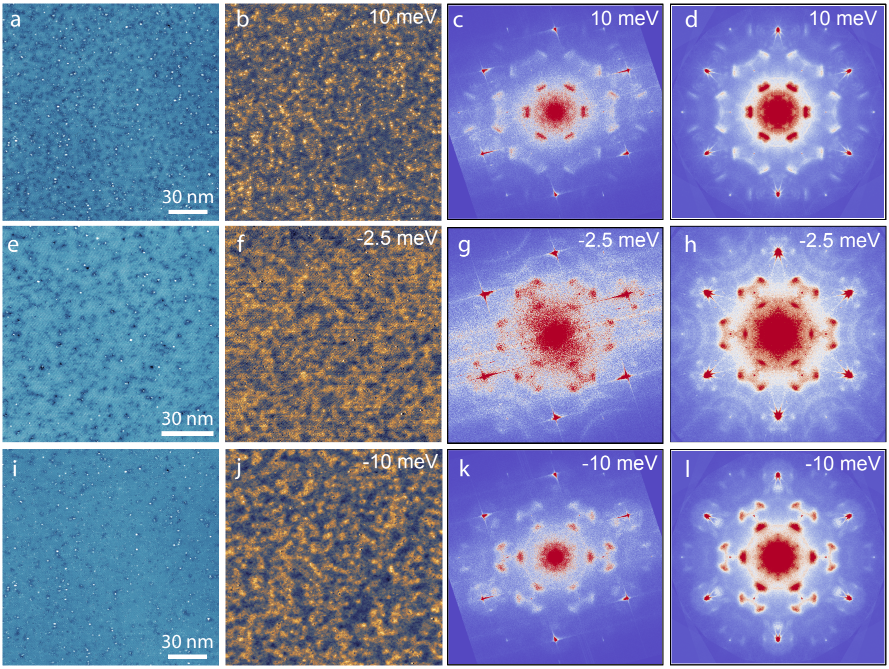

Supplementary Fig. 8: Symmetrization procedure for FT-STS images. (**a,e,i**),(**b,f,j**),(**c,g,k**), and (**d,h,l**), Topography, $dI/dV(r,V)$, FT of $dI/dV(r,V)$ and symmetrized FT results at different energies, respectively. Scanning drift has been corrected for (**c,g,k**). A horizontal line artifact feature in (g) has been intentionally suppressed by smoothing operation of nearby pixels before symmetrization.

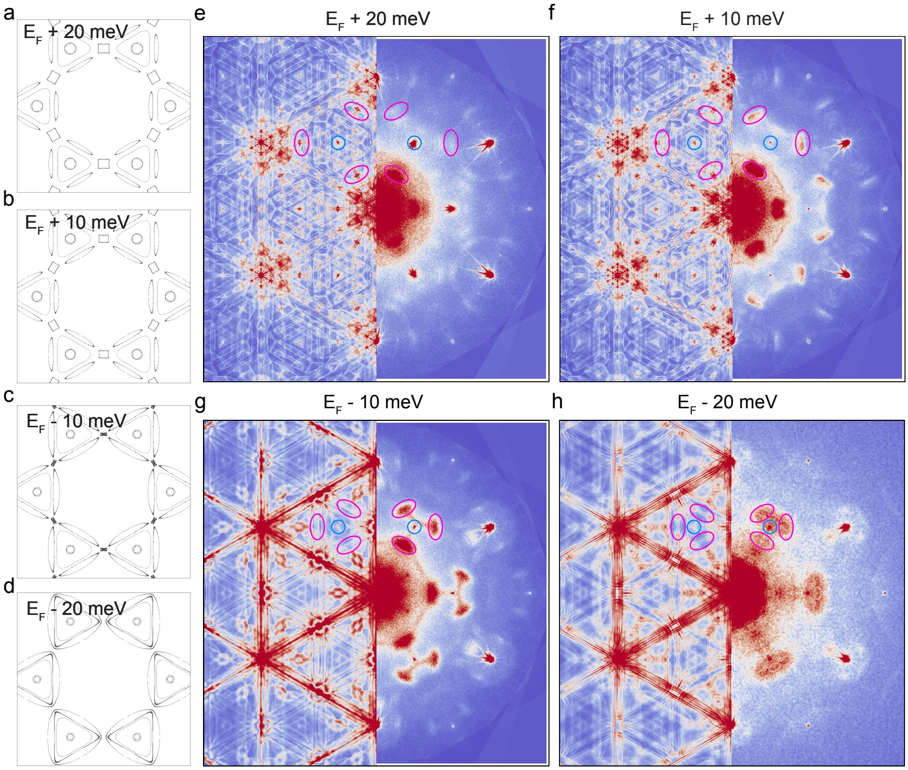

Supplementary Fig. 9: Comparison between the normal state JDOS and QPI images. a-d, Calculated constant energy contours of the band structure. **e - h**, Comparison between normal state JDOS (left) and experimental QPI images (right). Pink ovals mark the QPI features at each energy. The normal state JDOS cannot recover the experimental QPI features.

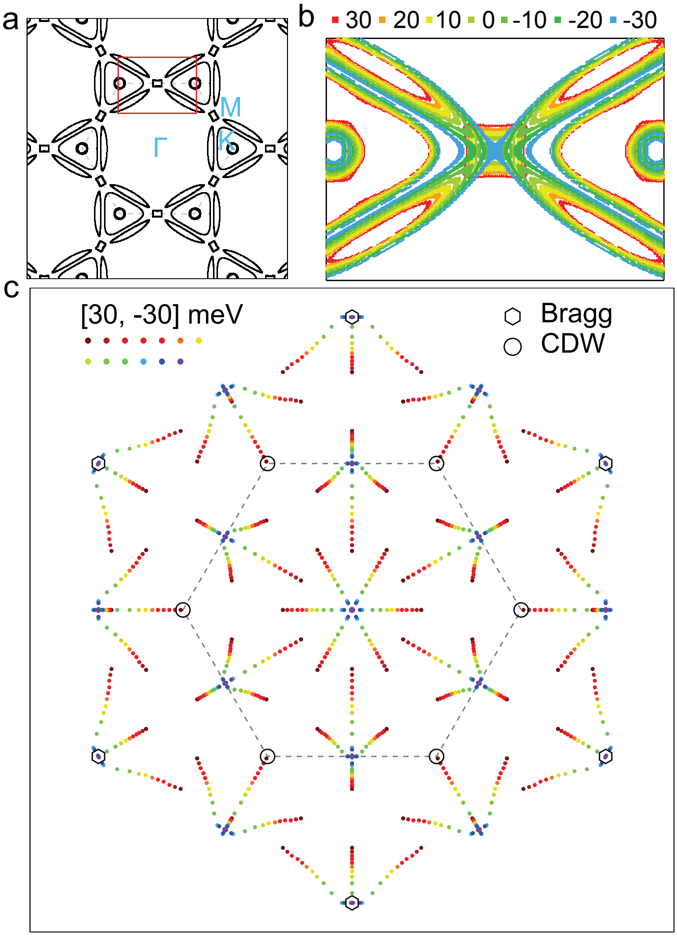

Supplementary Fig. 10: Simulation of scattering wavevectors in the normal state hot-spot model. a, Fermi surface by DFT calculation. **b**, Zoom-in image showing the dispersion of the bands near vHS (M point) in the highlighted region of (a). While multiple hot-spots exist in the bandstructure, only the α-band derived features can match the magnitude of the wavevector dispersion as a function of energy, while other features disperse much more slowly. **c**, Dispersion of all the α band scattering wavevectors in the hot-spot model.

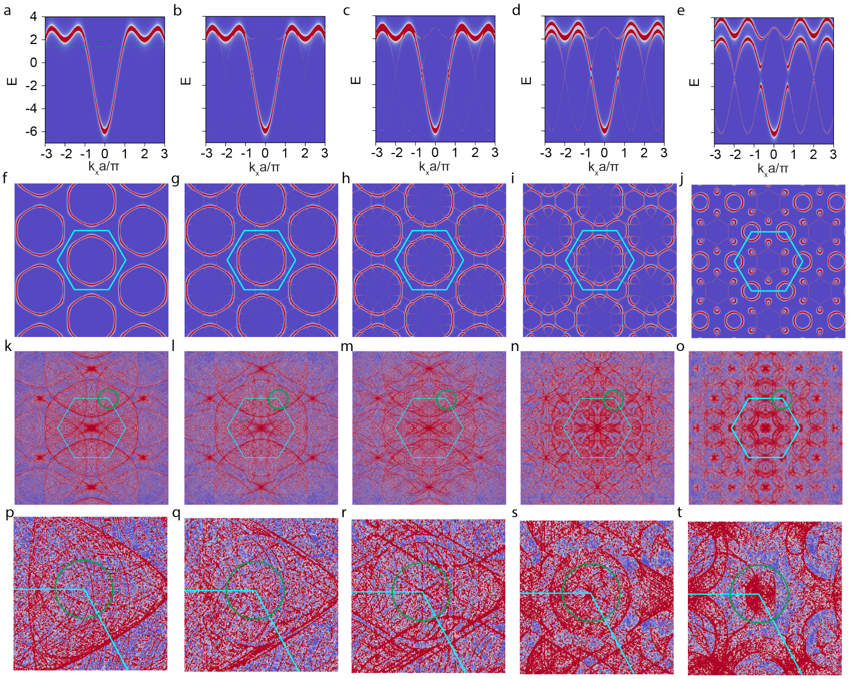

Supplementary Fig. 11: CDW lattice distortion and its coupling to the electronic spectrum. a-e, band dispersion the along $\Gamma - K$ direction, **f-j**, Constant energy contours at $\mu = 1.4t$ (marked by the dashed green line in (**a**)), **k-o**, JDOS, and, **p-t** zoom-in JDOS around $\mathbf{K}_{\text{CDW}}$ for different coupling constant values ($\frac{\lambda\Delta}{t} = 0, 0.05, 0.1, 0.2, 0.5$), respectively. The CDW wave-vector is (1/3, 1/3). The band dispersion is given by Eq. (9). The band reconstruction effects (spectral weight for the folded band and gap opening) become more visible as the coupling strength is increased. Intensity of the QPI patterns around $\mathbf{K}_{\text{CDW}}$ (region enclosed by the circles) becomes more pronounced as well. Blue hexagons mark the first Brillouin zone boundary.
